# Crippled Children

**Published:** 1920-05-08

**Authors:** 


### THE PUBLIC WELL-BEING.

### CRIPPLED CHILDREN.

### A National Scheme of Education and Care.

It is extraordinary how few people realise the very considerable number of crippled children that exist, or how great are the needs for organised care of them. The Central Committee for the Care of Cripples, acting in association with the Council of Infant and Child Welfare, has been giving close study to the subject, and has formulated a national scheme which we are enabled to publish. It has, first of all, set out by collecting the necessary information which hitherto has been most inadequate. With the exception of certain places, the provision of treatment and education for crippled children is wholly insufficient throughout the country, and no accurate figures are available to show the exact number of such children. In 1911 a partial survey was made in Birmingham, taking only those children known to social agencies; this gave the following return:

Population, 500,000.
Cripples over sixteen years of age, 1,001, or 2 per 1,000.
Under sixteen, 728, or 1.4 per 1,000.
Total, 1,729, or 3.4 per 1,000.

In 1918 an inquiry was made by the Joint Parliamentary Advisory Committee to demonstrate the need for the physically defective clause in the Education Act. This showed that, at that time, only fifteen authorities in England and Wales provided schools for the physically defective. There were in all sixty-seven of these schools, and London provided forty-one of them. London then had a school population of 740,000, of whom 3,700, or five per thousand, were attending physically defective schools. Glasgow provided for 1,790 such children. For that report returns were asked for from most of the big towns. Many replied that no figures were available, and others gave such returns as:

Population, 120,000—physically defective children, 20.
Population, 291,000—physically defective children, 15.
Population, 89,000—physically defective children, 10.
Population, 52,000—physically defective children, 8.

This clearly indicated that the physically defective children were not known to the local authorities.

A census taken recently in Cleveland, America, by house-to-house inquiry, gave:

Population, 674,073.
Cripples over fifteen, 3,265, or 4.7 per 1,000.
Under fifteen, 921, or 1.3 per 1,000.
Total, 4,186, or 6 per 1,000.

A census taken in Germany gave these facts:

Under fourteen, 1.4 per 1,000.
Total number under fourteen, 98,263.
Needing treatment, 56,320.
Beds available, 3,125.

Seven thousand children of school age were getting no schooling, although 91 per cent. of these were sound mentally.

There is a very special need for education for these children to enable them to compensate for their physical disabilities, and many of them have talents and are quick and clever. In regard to the need for treatment, Sir Robert Jones (Chairman of the Central Committee) says: "Children suffering from crippling diseases and deformities of all kinds exist in large numbers throughout the country. . . . The number of adult cripples is a still more common object for sympathy. Perhaps three-fourths of them could have been cured, and most of the others given much greater activity if their condition had been recognised early and treated efficiently." Treatment is lacking owing to the rarity of special hospitals, and the impossibility of providing effective treatment at general hospitals, owing to bed pressure.

The Central Committee for the Care of Cripples was formed from representatives of the surgeons who recognise the great need for more facilities for treatment, representatives from the Joint Parliamentary Advisory Committee, and representatives of the Invalid Children's Aid Association. The objects of the Committee are thus described:

(1) To promote a national scheme for complete provision of treatment and education for physically defective children throughout the country.

(2) To help any local authority or voluntary organisation desirous of making better provision for these children.

(3) To provide a central bureau of information on these matters.

(4) To act as a central co-ordinating body.

For this purpose it is necessary to get a clear idea of the total number of crippled children in England and Wales. The censuses taken in detail in Cleveland and in Germany show that the total number is large in those countries. The committee is carrying out a house-to-house inquiry in typical rural and industrial areas in order to obtain accurate figures per thousand population from which the national number can be estimated. It is also getting a complete return of schools and institutions for cripples, so as to ascertain to what extent adequate provision is already made.

A national scheme has been outlined which takes care not to interfere with existing efforts. Its chief points are:

I. A central co-ordinating body which should work with the Ministry of Health and the Board of Education.

II. Local committees for the care of cripples in every district.

III. The provision in England and Wales of an adequate number of hospital schools for crippled children with open-air wards.

**THE PUBLIC WELL-BEING—(*continued*).**

IV. Each hospital school should be run under its own local committee, with its own teaching staff and handicraft workshops.

Working in direct union with the hospital schools should be:

(*a*) Out-patient clinics for

(1) The examination of children sent up for consultation.

(2) The treatment of cases that can be effectively treated as out-patient.

(3) The after-treatment and supervision of children discharged from the hospital school.

(4) The maintenance of touch with old patients, so that advice and help may be given as to suitable employment.

(*b*) Invalid schools, day and residential,

For the minority of children who have not been cured, or benefited sufficiently to enable them to attend ordinary classes. In large towns special day schools will fill the need, but for children in other districts who are prevented from receiving education by their persisting disability, residential schools are necessary. For the sake of simplicity and economy these should be part of the establishment of the hospital school, but in a separate wing. These children would be drafted into this when fit to leave the hospital, but would continue to benefit from the teaching, handicraft shop, and surgical supervision.

V. It is vital that those responsible for the treatment should be experienced in orthopædic work. This applies both to the surgical staff and the hospital and after-treatment sisters.

VI. There should be provision for paying patients.

VII. Wards should be available for adolescent and adult cripples, and persons suffering from injuries needing orthopædic treatment.

VIII. Where efficient organisations for crippled children exist, these would be welcomed in the scheme which would be sufficiently elastic to cover some diversity of method.

The Ethel Hedley Hospital at Windermere, which was used for officer-patients needing orthopædic treatment during the war and is equipped for this work, has been transferred for the use of crippled children in accordance with this scheme, by the great kindness of Mr. Hedley, who pays all the expenses of running the hospital. The central committee also considers the present time offers a unique opportunity, because the hospitals set up by the Ministry of Pensions will cease to be required, and might be taken over by the Ministry of Health.

